# Quality of life, patient satisfaction, and cardiovascular outcomes of the randomised 2 x 3 factorial Copenhagen insulin and Metformin therapy (CIMT) trial – A detailed statistical analysis plan

**DOI:** 10.1016/j.conctc.2023.101095

**Published:** 2023-02-24

**Authors:** Markus Harboe Olsen, Thomas P. Almdal, Sten Madsbad, Christian Ovesen, Christian Gluud, Simone B. Sneppen, Leif Breum, Christoffer Hedetoft, Thure Krarup, Louise Lundby-Christensen, Elisabeth R. Mathiesen, Michael E. Røder, Henrik Vestergaard, Niels Wiinberg, Janus C. Jakobsen

**Affiliations:** a Copenhagen Trial Unit, Centre for Clinical Intervention Research, Copenhagen University Hospital − Rigshospitalet, The Capital Region, Copenhagen, Denmark; b Department of Neuroanaesthesiology, The Neuroscience Centre, Copenhagen University Hospital − Rigshospitalet, Copenhagen, Denmark; c Department of Endocrinology, Centre for Cancer and Organ Diseases, Copenhagen University Hospital − Rigshospitalet, Copenhagen, Denmark; d Department of Endocrinology, Copenhagen University Hospital − Amager and Hvidovre, Copenhagen, Denmark; e Department of Neurology, Copenhagen University Hospital − Bispebjerg and Frederiksberg, Copenhagen, Denmark; f Department of Regional Health Research, The Faculty of Health Sciences, University of Southern Denmark, Odense, Denmark; g Section of Endocrinology, Department of Internal Medicine, Copenhagen University Hospital − Herlev and Gentofte, Copenhagen, Denmark; h Department of Medicine and Endocrinology, Zealand University Hospital, Køge, Denmark; i Steno Diabetes Centre Zealand, Holbæk, Denmark; j Department of Clinical Medicine, University of Copenhagen, Copenhagen, Denmark; k Steno Diabetes Center Odense, Odense University Hospital, Odense, Denmark; l Department of Medicine, Bornholms Hospital, Rønne, Denmark; m Novo Nordisk Foundation Center for Basic Metabolic Research, University of Copenhagen, Copenhagen, Denmark; n Department of Clinical Physiology and Nuclear Medicine, Copenhagen University Hospital − Bispebjerg and Frederiksberg, Copenhagen, Denmark

**Keywords:** Type 2 diabetes, Metformin, Insulin, Randomised clinical trial, Clinical outcomes, Quality of life, Detailed statistical analysis plan

## Abstract

**Background:**

The evidence on the effects of metformin and insulin in type 2 diabetes patients on quality of life, patient satisfaction, and cardiovascular outcomes is unclear.

**Methods:**

The Copenhagen Insulin and Metformin Therapy (CIMT) trial is an investigator-initiated multicentre, randomised, placebo-controlled trial with a 2 × 3 factorial design conducted at eight hospitals in Denmark. Participants with type 2 diabetes were randomised to metformin (n = 206) versus placebo (n = 206); in combination with open-label biphasic insulin aspart one to three times daily (n = 137) versus insulin aspart three times daily in combination with insulin detemir once daily (n = 138) versus insulin detemir once daily (n = 137).

We present a detailed description of the methodology and statistical analysis of the clinical CIMT outcomes including a detailed description of tests of the assumptions behind the statistical analyses. The outcomes are quality of life (Short Form Health Survey (SF-36)), Diabetes Medication Satisfaction Questionnaire, and Insulin Treatment Satisfaction Questionnaire (assessed at entry and 18 months after randomisation) and cardiovascular outcomes including time to a composite of either myocardial infarction, stroke, peripheral amputation, coronary revascularisation, peripheral revascularisation, or death.

**Discussions:**

This statistical analysis plan ensure the highest possible quality of the subsequent post-hoc analyses.

**Trial registration:**

The protocol was approved by the Regional Committee on Biomedical Research Ethics (H-D-2007-112), the Danish Medicines Agency (EudraCT: 2007-006665-33 CIMT), and **registered** within ClinicalTrials.gov (NCT00657943, 8^th^ of April 2008).

## Teaser

An updated literature search 28th of January 2022 did not identify any additional randomised clinical trials evaluating the effects of adding metformin to insulin in people with type 2 diabetes. Therefore, the CIMT trial is the hitherto largest and longest randomised clinical trial assessing the effects of metformin in addition to insulin in people with type 2 diabetes on quality of life, well-being, and development of cardiovascular complications.

## 1 Background

The Copenhagen Insulin and Metformin Therapy (CIMT) trial is an investigator-initiated multicentre, randomised, placebo-controlled superiority trial with a 2 × 3 factorial design [1]. The CIMT trial evaluated the effect of an 18-month treatment with metformin versus placebo and simultaneously the effect of three insulin analogue regimens on the progression of mean carotid intima media thickness in patients with type 2 diabetes [1]. The CIMT trial was conducted from May 2008 to December 2012 and the trial results in relation to changes in carotid intima media thickness have been reported [2,3]. Metformin in combination with insulin did not reduce the carotid intima media thickness [2]; nor did the three insulin regimens [3]. Multiple exploratory analyses have since been carried out, but the prespecified patient-centred clinical outcomes quality of life, patient satisfaction, and time to a number of clinical and cardiovascular outcomes have not yet been reported [1,[4], [5], [6]].

One previous systematic review assessed the effects of adding metformin to insulin in people with type 2 diabetes [7]. We identified only three trials assessing the influence of metformin versus placebo on health-related quality of life or well-being [[7], [8], [9], [10]]. All three included trials were at high risk of bias, and all three trials had a shorter duration and included fewer participants than the CIMT trial [7]. None of the included trials reported significant differences regarding the patient-reported outcomes [[7], [8], [9], [10]]. Only Douek et al. [8] reported results of quality of life assessments in a way suitable for a meta-analysis, and it was therefore not possible to meta-analyse quality of life as an outcome [7]. The reporting of cardiovascular complications for the comparison of combining metformin with insulin compared with insulin monotherapy in people with type 2 diabetes was infrequent, and when meta-analyses could be performed, the data were sparse and effect estimates were non-significant [7].

Here, we describe a detailed statistical analysis plan of post-hoc analyses assessing the effects of metformin in addition to insulin and the effect of three different insulin regimens on patient-reported outcomes and cardiovascular complications in the CIMT trial.

## 2 Methods

**Fig. 1:**
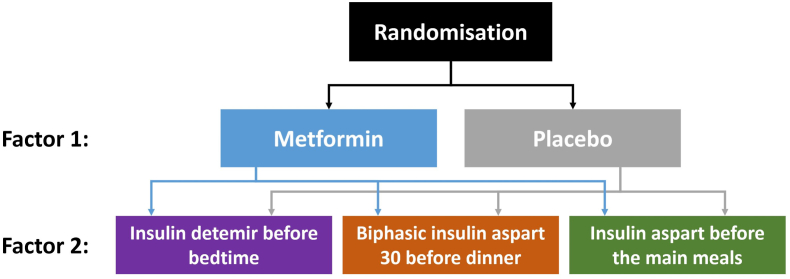
**–** A study scheme of the factorial designed trial, where participants are ranodmised into both factors. Thus, each participant is allocated in both Factor 1 and Factor 2, and receives a combination of the two treatment schemes.

The methodology of the CIMT trial have previously been published [1]. The CIMT trial participant inclusion criteria were: type 2 diabetes; age >30 years; body-mass index >25 kg/m^2^; HbA1c ≥ 7.5% (58 mmol/mol); and treatment with oral glucose-lowering drugs for at least one year and/or insulin treatment for at least three months [1]. After a screening visit, participants were centrally randomised at the Copenhagen Trial Unit according to the 2 × 3 factorial design. Participants were randomised 1:1:1 to treatment with one of three insulin analogue regimens and in a factorial way randomised 1:1 to treatment with metformin versus placebo (Fig. 1). Randomisation was stratified by age >65 years (yes/no), insulin treatment at trial entry (yes/no), and treatment at Steno Diabetes Center (yes/no) [1]. The insulin treatment was open-labelled, whereas participants, investigators, and medical staff were blinded to the metformin and placebo intervention (numbered identical containers) [1].

The protocol was approved by the Regional Committee on Biomedical Research Ethics (H-D-2007-112) and the Danish Medicines Agency (EudraCT: 2007-006665-33 CIMT), registered within ClinicalTrials.gov (NCT00657943), and conducted in accordance with The Declaration of Helsinki and guidelines for Good Clinical Practice. Informed consent was obtained from each participant enrolled in the trial.

The design of the CIMT trial and the influence of metformin and insulin on the primary outcomes have previously been reported [2,3]. Here we report the detailed statistical analysis plans for evaluating the effects of these intervention on the patient-centred outcomes quality of life, patient satisfaction, and cardiovascular outcomes.

We plan on reporting the results of these outcomes in two separate papers: one comparing metformin versus placebo comparison and one comparing the three insulin analogue regiments.

## 3 Outcomes

### 3.1 Quality of life and patient satisfaction

• Short Form Health Survey (SF-36) [11]. SF-36 is questionnaire and a general assessment of quality of life measures. Both a physical component score and a mental health component score are reported [11].
• Insulin Treatment Satisfaction Questionnaire (ITSQ) [12].
• Diabetes Medication Satisfaction Questionnaire (Diab-Medsat) [13].

These continuous outcomes were assessed at 18 months after randomisation [1].

### 3.2 Cardiovascular outcomes

• Time to myocardial infarction, stroke, peripheral amputation, coronary revascularisation, peripheral revascularisation, or death [1].
• Time to myocardial infarction, stroke, peripheral amputation, or death [1].
• Time to cardiovascular mortality [1].

The cardiovascular outcomes were assessed during the full observation period from randomisation of each patient into the trial and until June 11th, 2013 [1].

The outcome assessors and the participants were blinded to metformin versus placebo but not to the three insulin regimens [1]. We used the Danish Central Civil Register and the National Patient Register to obtain data on the cardiovascular outcomes [1].

## 4 Sample size and power considerations

Based on the sample size calculation in relation to the primary outcomes of the trial (change in mean intima media thickness of the common carotid arteries), we planned to include a total of 950 patients in the trial [1]. However, the trial was stopped due the end of the financing grant after the inclusion of a total of 412 participants [2,3]. For this factorial trial, this means that there is a comparison on 206 participants on metformin versus 206 participants on placebo and another comparison of three groups on different insulin regimens each including 137, 137, and 138 participants.

To increase the interpretability of the trial results and decrease the risks of random errors [14], we present power estimations for all planned outcomes [15]. We originally adjusted our threshold for statistical significance (the alpha value used in the sample size estimations) to 0.01 because of the multiple comparisons [1]. To take into account that the CIMT trial was stopped early (approximately halfway), we will now use an alpha value of 0.001 for the power estimations [14]. The following power estimations are based on the total 412 randomised participants for the metformin versus placebo comparison and a total of 274 randomised participants for each of the three pairwise insulin comparisons.

### 4.1 Short Form Health Survey (SF-36)

We consider 4 points as the minimal clinically relevant difference for SF-36 [14,[16], [17], [18], [19], [20], [21]], an alpha of 0.001, and a standard deviation (SD) of 9.5 points [12]. For the metformin versus placebo comparison, we will have a power of 83.0% to confirm or reject a 4-point increase on the SF-36. For each of the three pairwise insulin comparisons, we will have a power of 56.3% to confirm or reject a 4-point increase on the SF-36.

### 4.2 Insulin Treatment Satisfaction Questionnaire (ITSQ)

We consider 4 points as the minimal clinically relevant difference for ITSQ [14,20], an alpha of 0.001, and a standard deviation (SD) of 9 points [22]. For the metformin versus placebo comparison, we will have a power of 88.3% to confirm or reject a 4-point increase on the ITSQ. For each of the three pairwise insulin comparisons, we will have a power of 63.7% to confirm or reject a 4-point increase on the ITSQ.

### 4.3 Diabetes Medication Satisfaction Questionnaire (Diab-Medsat)

We consider 4 points as the minimal clinically relevant difference for Diab-Medsat [14,20,23], an alpha of 0.001, and a standard deviation (SD) of 9 points [24]. For the metformin versus placebo comparison, we will have a power of 88.3% to confirm or reject a 4-point increase on the Diab-Medsat. For each of the three pairwise insulin comparisons, we will have a power of 63.7% to confirm or reject a 4-point increase on the Diab-Medsat.

### 4.4 Time to myocardial infarction, stroke, peripheral amputation, coronary revascularisation, peripheral revascularisation, or death

We consider a 29% risk of experiencing one or more of the following outcomes: non-fatal myocardial infarction, stroke, peripheral amputation, coronary revascularisation, peripheral revascularisation, or death in the placebo group [25], an alpha of 0.001, and a hazard ratio reduction of 30%. For the metformin versus placebo comparison, we will only have 9.0% power to confirm or reject a hazard ratio reduction of 30%. For each of the three pairwise insulin comparisons, we estimate that we will only have 4.5% power to confirm or reject a hazard ratio reduction of 30%.

### 4.5 Time to the first of the following clinical outcomes myocardial infarction, stroke, peripheral amputation, or death

We consider a 12.5% risk of experiencing one or more of the following outcomes: non-fatal myocardial infarction, stroke, peripheral amputation, or death in the placebo group [25], an alpha of 0.001, and a hazard ratio reduction of 30%. For the metformin versus placebo comparison, we will only have 2% power to confirm or reject a hazard ratio reduction of 30%. For each of the three pairwise insulin comparisons, we estimate that we will only have 1.2% power to confirm or reject a hazard ratio reduction of 30%.

### 4.6 Time to cardiovascular death

We consider a 3.8% risk of experiencing death from cardiovascular disease in the placebo group [25], an alpha of 0.001, and a hazard ratio reduction of 30%. For the metformin versus placebo comparison, we will have almost 0% power to confirm or reject a hazard ratio reduction of 30%. For each of the three pairwise insulin comparisons, we estimate that we will have 0% power to confirm or reject a hazard ratio reduction of 30%.

We plan to present the results of these three cardiovascular outcomes in the Supplemental material because of the low statistical power.

## 5 Stratification and design variables

We used three stratification variables in the randomisation: age >65 years (yes/no), insulin treatment at trial entry (yes/no), and treatment at Steno Diabetes Center (yes/no) [1].

Other design variables were sex (male/female), prior cardiovascular disease (yes/no), statin treatment at entry (yes/no), and glutamic acid decarboxylase antibodies at entry (positive/negative) [1].

## 6 Definition of populations the following populations will be analysed

• A modified intention-to-treat population. This population includes all randomised participants except randomised participants who did not fulfil the inclusion criteria; randomised participants fulfilling one or more exclusion criteria; or randomised participants who did not receive any of the planned interventions [1].
• A per-protocol population. This population includes all those included in the modified intention-to-treat population, except participants who did not attend more than four out of the planned six visits following randomisation [1].

## 7 Handling of missing data

We will handle missing data according to the recommendation by Jakobsen and colleagues [26]. In short, we will consider using multiple imputation if it is not valid to ignore missing data [26]. If multiple imputation is used, then the primary result of the trial will be based on these data [26]. To take account of the possibility that data may be ‘missing not at random’, we will use a best-worst and worst-best case scenario as sensitivity analyses, which will assess the potential impact of the missing data on the trial results [26]. In the ’best-worst case’ scenario it is assumed that all patients lost to follow-up in the experimental group have had a beneficial outcome; and all those with missing outcomes in the control group have had a harmful outcome [26]. Conversely, in the ’worst-best case’ scenario, it is assumed that all patients who were lost to follow-up in the experimental group have had a harmful outcome; and that all those lost to follow-up in the control group have had a beneficial outcome [26]. When continuous outcomes are analysed, a ‘beneficial outcome’ will be defined as the group mean plus two SDs of the group mean, and a ‘harmful outcome’ will be defined as the group mean minus two SDs of the group mean for ‘best-worst case’ imputation [26].

When assessing quality of life and patient satisfaction, we will in secondary analyses assign the value ‘0’ for participants who died during the 18-months trial period.

We do not expect any missing values for the time to a cardiovascular outcome as all deaths and admissions to Danish hospitals are registered in the Civil Registry and the National Patient Registry and were adjudicated for clinical events by the blinded adjudication committee [2].

We will use the three stratification variables used in the randomisation and the four predefined design variables during the multiple imputation procedure to estimate the missing values, i.e. age >65 years (yes/no), insulin treatment at trial entry (yes/no), and treatment at Steno Diabetes Center (yes/no) as well as sex (male/female), prior cardiovascular disease (yes/no), statin treatment at baseline (yes/no), and glutamic acid decarboxylase antibodies (positive/negative) [26].

## 8 Statistical analysis

### 8.1 Thresholds for statistical and clinical significance

We will assess if the thresholds for statistical significance and clinical significance are crossed using the five-step procedure as suggested by Jakobsen and colleagues [14]. This procedure will include adjustments of thresholds for significance according to the number of outcome comparisons and the number of randomised participants in relation to the planned sample size [14]. Accordingly, we will analyse all continuous outcomes using Trial Sequential Analysis to take into account the early stopping of the trial [14,27]. When analysing the results using Trial Sequential Analysis, we will use the minimal important differences and the SDs defined in the paragraph above (see ‘Sample size and power considerations’), an acceptable risk of type I error of 1%, and an acceptable risk of type II error of 20% [1,14]. We will use a Bayes factor threshold for significance of 0.1 based on the a priory anticipated intervention effect [14].

### 8.2 Analysis of quality of life and patient satisfaction

Primary analysis: linear regression adjusted for the baseline value and the three stratification variables age >65 years (yes/no), insulin treatment at trial entry (yes/no), and treatment at Steno Diabetes Center (yes/no) [1].

Secondary analysis: linear regression adjusted for the baseline value and the stratification variables (see above) and the design variables (sex (male/female), prior cardiovascular disease (yes/no), statin treatment at entry (yes/no), and glutamic acid decarboxylase antibodies (positive/negative)) [1]. We will in a supplementary analysis adjust for metformin administration before trial entry (yes/no).

All analyses will primarily include the modified intention-to-treat population. We will also perform the analyses adjusted for the stratification variables including the per-protocol population [1].

### 8.3 Analysis of the cardiovascular outcomes

*Primary analysis:* Cox regression analysis adjusted for the three stratification variables (age >65 years (yes/no), insulin treatment at trial entry (yes/no), and treatment at Steno Diabetes Center (yes/no)) [1].

*Secondary analysis:* Cox regression analysis adjusted for stratification variables (see paragraph above) and other design variables (sex (male/female), prior cardiovascular disease (yes/no), statin treatment at entry (yes/no), and glutamic acid decarboxylase antibodies (positive/negative)) [1]. We will in a supplementary analysis adjust for metformin administration before trial entry (yes/no).

### 8.4 Assessments of underlying statistical assumptions

We plan to systematically assess if the assumptions underlying each of the used statistical methods are fulfilled [28].

For all analyses, we will test for major interactions between each covariate and the intervention variable (test of interaction). In turn, we will include each possible first order interaction between included covariates and the intervention variable. For each combination, we will test if the interaction term is significant and assess the effect size. We will only consider that there is evidence of an interaction if the interaction is statistically significant after Bonferroni adjusted thresholds (0.05 divided by number of possible interactions) and if the interaction shows a clinically significant effect. If it is concluded that the interaction is significant, we will consider both presenting an analysis separately for each (e.g. for each site if there is significant interaction between the trial intervention and ‘site’) and an overall analysis including the interaction term in the model.

### 8.5 Assessments of underlying statistical assumptions for linear regression

We will visually inspect quantile-quantile plots of the residuals [28,29] to assess if the residuals are normally distributed and use residuals plotted against covariates and fitted values [28,29] to assess for homogeneity of variances. If the plots do not show a straight line, we will consider transforming the outcome, e.g. using log transformation or square root and/or use robust standard errors [28,29].

### 8.6 Assessments of underlying statistical assumptions for cox regression

We will visually inspect log-log plots stratified by treatment and adjusted for the effects of all covariates (continuous and categorical) to asses if the assumption of proportional hazards between the compared intervention groups is fulfilled [[28], [29], [30]]. If the assumption of proportional hazards seems violated, we will consider stratifying the regression analysis for the variable not fulfilling the assumption or using a non-parametric test (e.g. log rank test) as the primary analysis method or split the observation period into two (or more) separate observation periods.

## 9 Statistical reports

Blinded data on all outcomes will be analysed by two independent investigators [28]. Two independent statistical reports will be sent to the CIMT Steering Committee, and if there are discrepancies between the two primary statistical reports, then possible reasons for that will be identified and it will be considered which is the most correct result [28]. A final statistical report will be prepared, and all three statistical reports will be published as supplementary material.

## 10 Characteristics of patients at baseline

We will present a description of baseline characteristics by intervention group. Discrete variables will be summarised by frequencies and percentages. Percentages will be calculated according to the number of patients with available data. Where values are missing, the actual denominator will be stated. Continuous variables will be summarised using standard measures of central tendency and dispersion using either mean ± SD for data with normal distribution or median and interquartile range for non-normally distributed data.

## 11 Deviations from the initial design and methods of the CIMT trial

Based on our sample size estimation for the primary outcome of the CIMT trial allowing for five comparisons of the involved intervention groups we planned to include 950 patients [1]. However, eventually only 412 participants were included, as the trial had to be stopped at the scheduled duration of the trial due to lack of financial support. In the present analysis plan we schedule to analyse these clinical outcomes based on a strict factorial 2 × 3 design comparing the effects of metformin versus placebo and comparing the three different insulin analogue regimens. Given the low power in relation to the clinical outcomes, we consider all outcomes to be exploratory and that most emphasis should be given to the quality of life and patient satisfaction results according to our result of the power calculations.

In the original protocol it was not specified how the patient-important quality of life data and clinical outcomes would be analysed and presented [1]. Given the fact that the trial is a 2 × 3 factorial design and two sets of data are presented, we anticipate publishing the data in two separate papers.

## 12 Discussion

To avoid risks of outcome reporting bias and data-driven biased results, this paper presents the detailed statistical analysis plan for the CIMT trial analysing patient-relevant outcomes at a time when the data have been gathered but not yet inspected or analysed. This statistical analysis plan follows the guidelines provided by Gamble et al. [31]. Clinical trials ought to be analysed according to a pre-specified analysis plan in order to prevent reporting bias and data driven analysis results [[32], [33], [34], [35]]. We have an obligation to report these patient-centred outcomes of quality of life, patient satisfaction, and cardiovascular outcomes and make the results available for future systematic reviews.

## 13 Strengths

This statistical analysis plan has a number of strengths. The overall trial methodology has been pre-defined, we take problems with multiplicity and other risks of random errors into account, use validated statistical methods, and we systematically test for the underlying statistical assumptions [1,14,28]. Furthermore, we will analyse data in accordance to the modified intention-to-treat principle and, if necessary, use multiple imputations, and a best-worst/worst-best case scenario to assess the potential impact of the missing data on the results [26]. We will use the five-step procedure proposed by Jakobsen and colleagues to assess whether the thresholds for statistical and clinical significance are crossed [14].

## 14 Limitations

The statistical analysis plan also has limitations. First, the primary outcome of the CIMT trial was an non-validated surrogate outcome [1], and the analyses of the patient-centred outcomes described in the present statistical analysis plan should therefore be considered exploratory. Second, the power estimations of the cardiovascular outcomes showed that we do not have sufficient data to confirm or reject realistic intervention effects [15], so the results of the cardiovascular outcome will only be reported in the supplementary material. Third, this statistical analysis plan has been developed long after data accrual was stopped. However, the plan was developed and agreed upon without knowledge of the intervention effects or specific data.

We plan on a subsequent 10-year follow-up study of the cardiovascular outcomes, assessed using similar methodology as described in this statistical analysis plan. This would compensate for the low power regarding the cardiovascular outcomes because of only 18 months follow-up in 412 patients, we plan to prolong the follow-up for the participants after end of the trial. This would raise the power of our analyses of cardiovascular outcomes, but risk confounding by changes to anti-diabetic treatment after stop of the CIMT interventions.

## 15 Conclusions

To avoid risks of outcome reporting bias and data-driven biased results, this article presents the detailed statistical analysis plan for the CIMT trial analysing patient-relevant outcomes at a time when the data have been gathered but not yet inspected or analysed.

## Funding

The investigators received an unrestricted grant from 10.13039/501100004191Novo Nordisk to enable conduct of the trial. The trial and its main 2 × 3 factorial design was 100% initiated and conducted by the investigators. Novo Nordisk was allowed to comment on the protocol, on protocol changes during the trial, and on the manuscript prior to submission.

## Author contributions and consent for publication

MHO, JCJ, CG, CO, and TA: Drafted the present manuscript and revised and approved the final version. All remaining authors contributed with: 1) substantial contribution to the conception and design of the work; 2) acquisition and interpretation of data; 3) critically revising the work for intellectual content; and 4) agreement to be accountable for all aspects of the work.

## Ethics approval and consent to participate

Central ethical approval has been confirmed from the Regional Committee on Biomedical Research Ethics (H-D-2007-112) and the Danish Medicines Agency (EudraCT: 2007-006665-33 CIMT), registered within ClinicalTrials.gov (NCT00657943), and the trial was conducted in accordance with The Declaration of Helsinki and guidelines for Good Clinical Practice. Informed consent was obtained from each participant enrolled in the trial.

## Consent for publication

Not applicable.

## Availability of data and material

All relevant data are available.

## Declaration of competing interest

*Sten Madsbad:* Advisory boards: AstraZeneca; Boehringer Ingelheim; Eli Lilly; Intarcia Therapeutics; Merck Sharp & Dohme; Novartis; Novo Nordisk; Sanofi. Lecture fees: AstraZeneca; Boehringer Ingelheim; Merck Sharp & Dohme; Novo Nordisk; Sanofi. Research Grant Recipient: Novo Nordisk; Boehringer Ingelheim. *Leif Breum:* Advisory boards: AstraZeneca; Boehringer Ingelheim; Merck Sharp & Dohme; Novo Nordisk; Sanofi. Lecture fees: AstraZeneca; Lundbeck, Otsuka. *Louise Lundby-Christensen, Christian Ovesen, Thomas Almdal:* own shares in Novo Nordisk A/S. *Elisabeth R Mathiesen:* Advisory board: Novo Nordisk. *Markus Harboe Olsen. Janus C Jakobsen, Christian Gluud, Simone B Sneppen, Christoffer Hedetoft, Michael E. Røder, Henrik Vestergaard, Niels Wiinberg, Thure Krarup:* none declared.

## Data Availability

No data was used for the research described in the article.
